# Antioxidant and Anti-Inflammatory Activities of Barettin 

**DOI:** 10.3390/md11072655

**Published:** 2013-07-22

**Authors:** Karianne F. Lind, Espen Hansen, Bjarne Østerud, Karl-Erik Eilertsen, Annette Bayer, Magnus Engqvist, Kinga Leszczak, Trond Ø. Jørgensen, Jeanette H. Andersen

**Affiliations:** 1 MabCent-SFI, University of Tromsø, Breivika N-9037 Tromsø, Norway; E-Mail: trond.jorgensen@uit.no; 2 Marbio, University of Tromsø, Breivika N-9037 Tromsø, Norway; E-Mails: espen.hansen@uit.no (E.H.); jeanette.h.andersen@uit.no (J.H.A.); 3 Department of Medical Biology, Faculty of Health Sciences, University of Tromsø, N-9037 Tromsø, Norway; E-Mail: bjarne.osterud@uit.no; 4 Faculty of Biosciences, Fisheries and Economics, University of Tromsø, N-9037 Tromsø, Norway; E-Mail: karl-erik.eilertsen@uit.no; 5 Department of Chemistry, University of Tromsø, N-9037 Tromsø, Norway; E-Mails: annette.bayer@uit.no (A.B.); magnus.engqvist@uit.no (M.E.); kinga.leszczak@uit.no (K.L.)

**Keywords:** anti-inflammatory, antioxidant, barettin, natural product

## Abstract

In this paper, we present novel bioactivity for barettin isolated from the marine sponge *Geodia barretti*. We found that barettin showed strong antioxidant activity in biochemical assays as well as in a lipid peroxidation cell assay. A de-brominated synthetic analogue of barettin did not show the same activity in the antioxidant cell assay, indicating that bromine is important for cellular activity. Barettin was also able to inhibit the secretion of the inflammatory cytokines IL-1β and TNFα from LPS-stimulated THP-1 cells. This combination of anti-inflammatory and antioxidant activities could indicate that barettin has an atheroprotective effect and may therefore be an interesting product to prevent development of atherosclerosis.

## 1. Introduction

**Figure 1:**
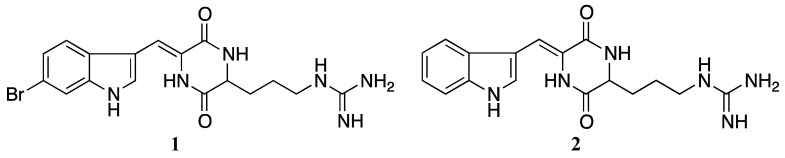
Barettin (**1**) and debromobarettin (**2**).

It is a well-known fact that sponges are rich sources of bioactive compounds. Barettin (cyclo-[6-bromo-8-en-tryptophan]-arginine]) was first isolated and described in 1986 [1] from the marine sponge *Geodia barretti* and in 2002, Sölter *et al.* published data suggesting a slight structure modification of the originally proposed molecule [2]. This revised structure (Figure 1(**1**)) was later confirmed by Johnson *et al.*, when they successfully synthesized barettin [3]. 

Previously, barettin was proven to have anti-fouling properties [4,5,6,7] and these properties are thought to be caused by the serotonin-like structure (Figure 1(**1**)) [8,9]. The identification of barettin from *G. barretti* collected outside Northern Norway was the result of the antioxidant screening project at MabCent-SFI [10]. The compound showed strong antioxidant activities in biochemical assays, and we decided to further investigate the possibility of new biological activities of this molecule. These studies included cellular antioxidant assays and anti-inflammatory assays. As the antioxidant defence system and the immune system are closely linked in diseases like arthritis, diabetes, asthma and coronary heart diseases, we were interested in examining whether barettin also has an anti-inflammatory effect. Several natural products which have combined antioxidant and anti-inflammatory effects have previously been described, especially from fruits and plants [11,12]. Of the more well-known are fucoxanthin and resveratrol. Fucoxanthin exerts many effects, including radical scavenging and inhibition of several inflammatory cytokines and mediators [13] whereas resveratrol induces antioxidant enzymes as well as reduce atherosclerotic lesions [14]. 

The purpose of this work was to study the antioxidant and anti-inflammatory effects of barettin. Originally, less than 13 mg barettin was isolated from *G. barretti*. In order to perform bioactivity studies and confirm the structure, we synthesized barettin and also a de-brominated analogue (Figure 1(**2**)), which were both tested in biochemical and cellular assays. Thus, in this paper we will present novel antioxidant and anti-inflammatory activities for barettin and debromobarettin.

## 2. Results and Discussion

### 2.1. Bioactivity Testing

#### 2.1.1. Antioxidant Activity

**Figure 2:**
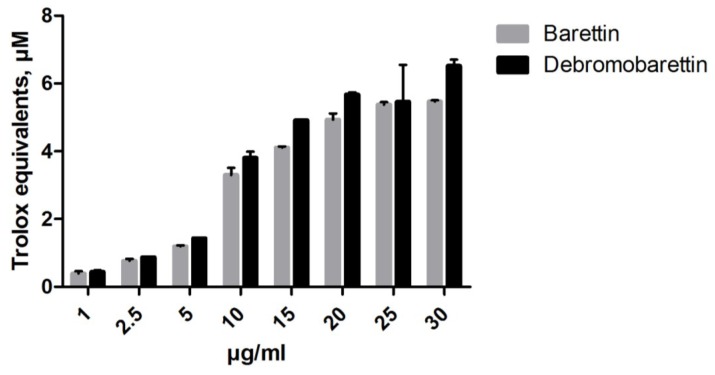
Both barettin and the debromobarettin act in a dose-dependent manner in the oxygen radical absorbance capacity (ORAC) assay to protect fluorescein from degradation (*n* = 2).

**Figure 3:**
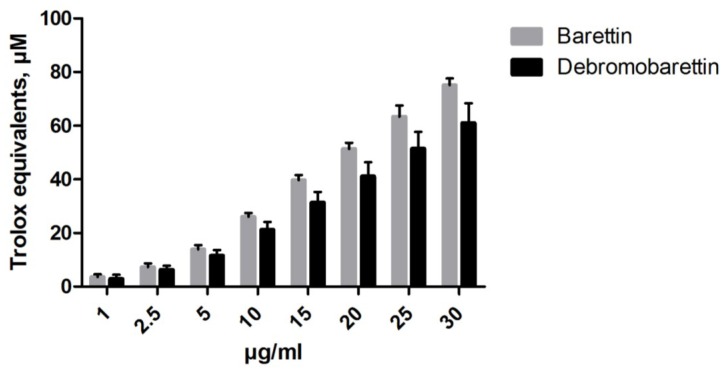
The antioxidant effect of barettin and debromobarettin tested in the ferric reducing antioxidant power (FRAP) assay. Both molecules reduce iron in a dose-dependent manner (*n* = 2).

We used the biochemical assays FRAP (ferric reducing antioxidant power) and ORAC (oxygen radical absorbance capacity) to obtain an indication of the antioxidant potential of barettin and the synthetic de-brominated analogue. A dose-response activity was observed for both compounds (Figure 2, Figure 3). At a concentration of 30 μg/mL (71.6 μM) barettin had a FRAP value of 77 μM trolox equivalents (TE) whereas the ORAC value was 5.5 μM TE. 

Biochemical assays are fast, cost-effective and can offer useful information of antioxidant activity. Cellular assays can offer more biological relevant information since they take into account the bioavailability and metabolism of the tested compound [15,16]. To further explore the antioxidant potential of barettin we tested the compounds in two cellular antioxidant assays using HepG2 cells, a human liver hepatocellular carcinoma cell line often used to study the antioxidant effect of natural products [17]. Murakami *et al.* for instance used HepG2 to study the effect of catechins on cellular antioxidant systems [18] and Alia *et al.* showed that quercetin could protect these cells from oxidative stress by *tert*-butyl hydroperoxide [19].

**Figure 4:**
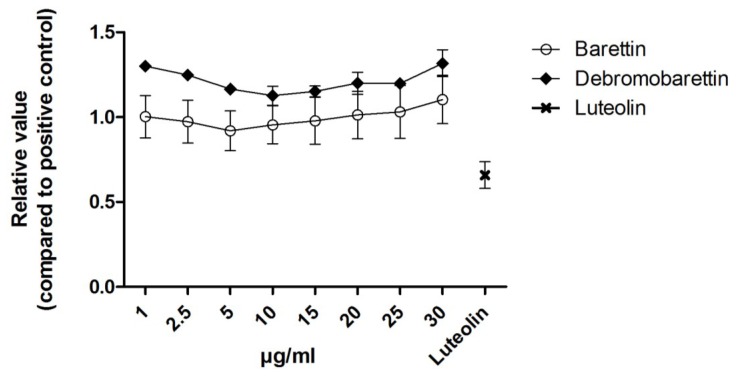
Cellular Antioxidant Activity (CAA) results for barettin and debromobarettin. Neither compound reduced the intracellular oxidation in HepG2 cells. Luteolin (10 μM) was used as a comparative control. Results are normalized to a positive control 2,2′-Azobis(2-methylpropionamidine) dihydrochloride (AAPH) (*n* = 3).

**Figure 5:**
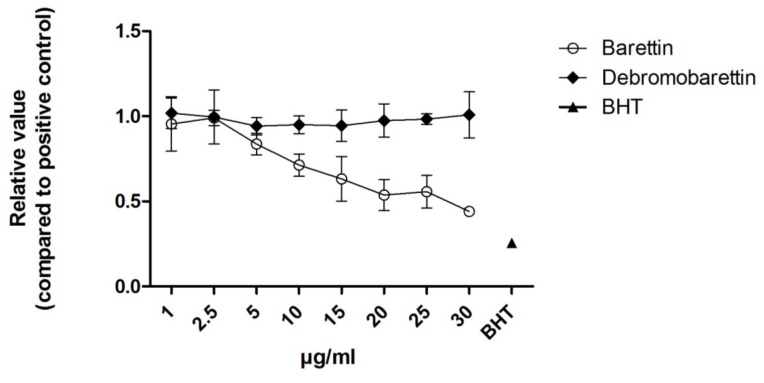
Cellular Lipid Peroxidation Antioxidant Activity (CLPAA) results for barettin and debromobarettin. Barettin acts in a dose-dependent manner to reduce lipid peroxidation in HepG2 cells. This effect was not seen with the de-brominated molecule. Butylated hydroxytoluene (BHT; 10 μM) was used as a comparative control. Results are normalized to positive control with cumene hydroperoxide (CumOOH) (*n* = 3).

We used the cellular antioxidant activity assay (CAA, Figure 4) and the cellular lipid peroxidation antioxidant activity (CLPAA, Figure 5) assays [20,21] to measure the intracellular reactive oxygen species (ROS) and the lipid membrane antioxidant activity, respectively. None of the molecules had any effect in the CAA assay (Figure 4). Barettin gave a 55% reduction in lipid peroxidation compared to the control in the CLPAA assay (Figure 5), whereas debromobarettin did not show any activity. 

The cellular assays give more information about the antioxidant activity where the CAA indicates the overall oxidative status of the cell by monitoring the decomposition of the water-soluble probe 2′,7′-Dichlorofluorescin diacetate (DCFH-DA) into the fluorescent dichlorofluorescein (DCF) [20,22]. The CLPAA assay on the other hand detects antioxidants preventing lipid peroxidation in cellular membranes by monitoring the increase of a green fluorescence product produced by the lipophilic probe C-11-BODIPY after addition of cumene hydroperoxide (CumOOH) [21].

The lack of response in the CAA assay and the inhibition observed in the CLPAA assay indicates that barettin is not able to prevent formation of the fluorescent DCF by reactive oxygen species (ROS) but inhibits radicals from oxidizing the C-11-BODIPY and membrane lipids. 

The barettin molecule has a polar arginine and a non-polar brominated tryptophan end (Figure 1(**1**)) [2,3]. The bromine present in barettin is the only feature distinguishing it from the de-brominated synthetic analogue (Figure 1). Several research groups have proved that halogens are important for cellular antioxidant activity such as Gentry *et al.* who studied the effect of inserting halogen atoms in the DPLPE-Phe enkephalin([D-Pen^2^-,L-Pen^5^,Phe^6^]) [23]. They observed that the lipophilicity and the cell membrane solubility of the CNS-acting drug are dependent on halogenation. Also Gerebtzoff *et al.* used parameters like surface activity and permeability coefficient to show that halogenation improves the drug membrane binding and diffusion in general [24]. Thus we believe that the absence of bromine in the de-brominated analogue reduces the bioavailability and explains the lack of inhibition of the lipid peroxidation seen in the HepG2 cells used in the CLPAA assay. 

Reactive oxygen species (ROS) and reactive nitrogen species (RNS) can oxidize lipids in low-density lipoprotein (LDL) and cell membranes and lead to conditions like atherosclerosis and chronic inflammation [25,26]. The oxidized lipids are recognized by pattern recognition receptors on immune cells and elicit an immune response by attracting monocytes [27]. In atherosclerosis, foam cell formation initiated by activated macrophages by uptake of oxidized LDL generate plaque development in the blood vessel intima [25]. In this scenario, antioxidants are thought to prevent LDL and cell membrane lipids from being oxidized and thus inhibit development of oxidative stress related diseases, including plaque development and atherosclerosis [28]. 

#### 2.1.2. Anti-Inflammatory Activity

**Figure 6:**
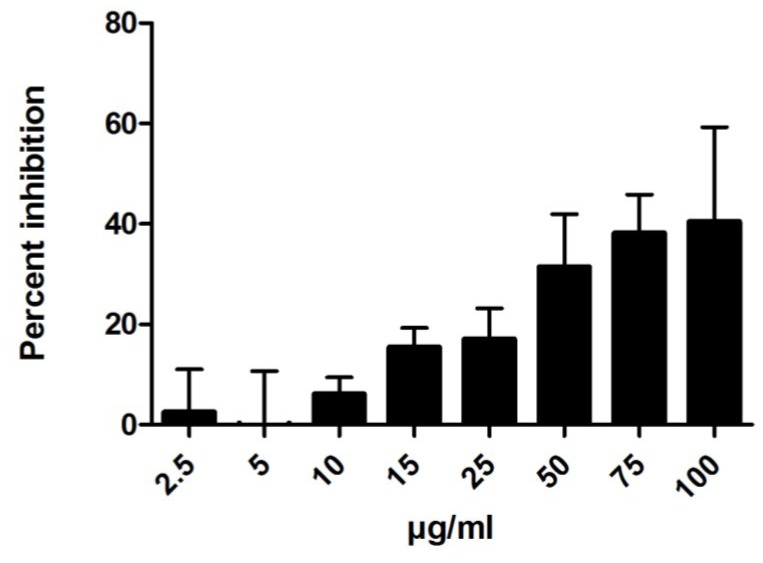
Interleukin-1β secretion from THP-1 cells were inhibited by barettin in a dose-dependent manner (*n* = 3).

Atherosclerosis is recognized as a chronic inflammatory disease where oxidative stress is involved in the onset and progression [29]. We wanted to study whether barettin could also have an anti-inflammatory effect in addition to the lipid peroxidation inhibition observed, making it highly interesting as a possible atheroprotective compound. Using the human acute monocytic leukemia cell line (THP-1) and ELISA, we monitored the tumor necrosis factor α (TNFα) and interleukin-1β (IL-1β) production. We found a dose-dependent inhibition of IL-1β production with increasing concentration of barettin (up to 100 μg/mL, Figure 6). At concentrations ranging from 50 to 100 μg/mL TNFα secretion was also inhibited (data not shown). 

As the anti-inflammatory cell system is very sensitive to endotoxin contaminants, LPS present in the environment as endotoxin can in even small amounts affect any bioassay [30,31]. It is therefore essential to avoid such contamination in immune assays. In order to obtain endotoxin-free samples we used polymyxin B based gel-packed columns to remove pyrogens. The pyrogen-free sample was then used in the anti-inflammatory assay. 

### 2.2. Cytotoxicity

The previously reported anti-fouling properties of barettin and the presented cellular effects could be caused by a general toxicity with the bromine as a cytotoxic “inducer”. Hepatocytes are good models for studying toxicity since the liver is the primary site for drug metabolism and biotransformation [32,33]. In addition to the hepatocyte cell line HepG2 we also included normal lung fibroblasts (MRC-5) and THP-1 cells when testing for cytotoxicity. 

**Figure 7:**
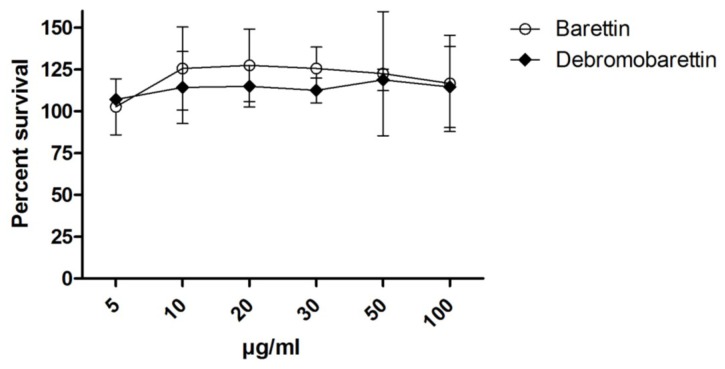
Results from cytotoxicity testing of barettin and debromobarettin using HepG2 cells**.** Results are expressed as percent survival after 24 h exposure (*n* = 2).

Cytotoxicity was tested using the CellTiter 96 AQueous One Solution Assay (Promega). In the CAA and CLPAA assays the cells were exposed to the test compounds for 1 h before washing. Thus a 2 h exposure on HepG2 cells should detect whether barettin and/or debromobarettin are likely to cause cell death and false results in these assays. The three cell lines were also exposed to the compounds for 24 h in a separate testing. This would reveal more long-term damage or whether any toxicity was caused by something other than membrane lysis. As can be seen from Figure 7, neither barettin nor debromobarettin were toxic to the HepG2 after 24 h in the concentrations tested. Toxicity was neither detected in THP-1 (after 6 and 24 h) nor MRC-5 cells (data not shown). 

Barettin was also tested for cytotoxicity on HepG2 and MRC-5 after 72 h exposure (data not shown), and the compound did not show cytotoxicity until concentrations reached 100 µg/mL, a concentration well above the maximum of 30 μg/mL used in the CAA/CLPAA assays.

## 3. Experimental Section

### 3.1. Purification, Isolation and Identification

The sponge was collected by bottom trawling at 390 m depth in the Barents Sea. The lyophilized material was extracted twice with ultrapure water (2 × 1000 mL) at 4 °C and centrifuged at 4500× *g* and 5 °C for 30 min before the pellet was freeze dried. The lyophilized pellet was extracted twice with 1000 mL dichloromethane:methanol (1:1, v:v) at 4 °C and subsequently filtered through a Whatman No3 filter. The filtrate was reduced to an orange oily liquid at 40 °C and reduced pressure in a rotary evaporator giving 9 g of organic extract. The organic extract was further chromatographed on a HP20-resin using a solvent step-gradient system of 5%, 25%, 50% and 75% aqueous methanol and two last steps of 100% methanol and 100% acetone. The fraction eluted with 50% methanol was reduced to dryness and dissolved in 1 mL 50% aqueous acetonitrile. Barettin was isolated using a Waters HPLC auto-purification system equipped with a Waters XTerra C18 column (10 × 300 mm, 10 µm) and eluted with a gradient from 25% to 35% of acetonitrile (ACN) and water, both containing 0.1% formic acid and at a flow rate of 6 mL/min. Two isomers of barettin eluted as two peaks giving 12.9 and 3.9 mg pure compound (retention time 5.1 and 6.3, respectively). High-resolution ESIMS gave *m/z* 419.0830 [M + H]^+^, as the calculated *m/z* for C_17_H_19_BrN_6_O_2_ ([M + H]^+^) is 419.0826.

### 3.2. Synthesis

Barettin and debromobarettin were synthesized according to the published procedure for barettin [3]. The (*L*)-form of *N*^α^-(*tert*-butoxycarbonyl)-*N*^ω^, *N*ω′-bis(*tert*-butoxycarbonyl)-arginine was used as starting material (Bachem, Switzerland). The ^1^NMR and HRMS data were in agreement with published data [2,5]. The crude synthetic products were purified on a Waters HPLC auto-purification system using a Waters XTerra C18 column (10 × 300 mm, 10 µm) and ACN and water (both containing 0.1% formic acid) as mobile phase at a flow rate of 6 mL/min. Barettin was isolated using a gradient from 20% to 40% ACN over 10 min (*R_t_* 7.0 min), and debromobarettin was isolated using a gradient from 15% to 25% ACN over 15 min (*R_t_* 7.2 min).

### 3.3. Biochemical Assays

FRAP assay. Reagents were prepared according to Benzie and Strain [34] and carried out in a DTX 880 Multimode Detector (Beckman Coulter, CA, USA) at 595 nm. Trolox (Sigma-Aldrich, St. Louis, MO, USA) was used to prepare the standard curve (0–250 μM; working concentration). The FRAP reagent (TPTZ (2,4,6-Tris(2-pyridyl)-*s*-triazine): Sigma-Aldrich; Fe: Merck, Darmstadt, Germany) was prepared daily. The assay was carried out in clear 96-well plates with 20 μL sample and 150 μL FRAP-reagent added to each well in duplicates. Water was used as a blank. Samples were incubated at 37 °C for 30 min before reading the plate. The blank was subtracted from each sample and a standard curve was created from the average absorbance of the duplicated trolox samples. The equation generated from the standard curve was used to calculate the trolox equivalents (TE) from each sample. Results were expressed as μM TE. 

ORAC assay. The method has been modified from Huang *et al.* [35]. The assay was carried out in black 96-well plates (Nunc) using a Victor3 Plate Reader (Perkin Elmer, MA, USA) at 37 °C (excitation 486 nm, emission 520 nm). All reagents were dissolved in 75 mM phosphate buffer (PB, pH 7.4). Diluted concentrations of barettin and debromobarettin were added in duplicates followed by addition of 125 μL fluorescein (52 nM final concentration, Sigma-Aldrich). After a 15 min incubation at 37 °C, 60 μL AAPH (2,2′-Azobis(2-methylpropionamidine) dihydrochloride; Sigma-Aldrich) was quickly added to each sample (44 mM final concentration). Fluorescence was recorded 25 times at 37 °C with a 70 s. delay between repeats. Trolox (0–25 μM working concentration) was included in each run to make a standard curve. PB was used as a blank and for the 0 μM Trolox sample. Area under the curve (AUC) was calculated by subtracting the AUC_Blank_ values. A standard curve was created using the trolox values and trolox equivalents of the samples were calculated from the resulting equation. Results were expressed as μM TE.

### 3.4. Cellular Assays

HepG2 and MRC-5 cells were grown in MEM Earle’s medium (F0325) supplemented with gentamycin (10 μg/mL), non-essential amino acids (1%), sodium pyruvate (1 mM), l-alanyl-l-glutamine (2 mM) and fetal bovine serum (FBS, 10%) and incubated at 37 °C with 5% CO_2_. Media and supplements were from Biochrom (Berlin, Germany). THP-1 cells were grown in RPMI-1640 (Biochrom) supplemented with gentamycin and FBS and incubated at 37 °C with 5% CO2. To differentiate the monocytes into macrophages, 50 ng/mL phorbol 12-myristate 13-acetate (PMA) (Sigma Aldrich) was added. 

#### 3.4.1. Cellular Lipid Peroxidation Antioxidant Activity (CLPAA) Assay

Approximately 80,000 HepG2 cells per well were seeded in black 96 well plates with clear bottom (Corning, NY, USA) and incubated overnight. Cells were washed with PBS between additions of new reagents. Total reaction volume was 100 μL. All incubations were carried out at 37 °C with 5% CO_2_. The cells were labelled with C11-BODIPY (Invitrogen, Eugene, OR, USA) for 30 min and incubated for 1 h with various concentrations of the test compounds. Cumene hydroperoxide (CumOOH, Sigma-Aldrich) was added to initiate lipid peroxidation and the plate was immediately installed in a Victor3 Plate Reader. Both red (590/7 nm (excitation), 632/45 nm (emission)) and green (485/14 nm, 520/10 nm) fluorescence were recorded. Percent inhibition was calculated relative to the positive control (CumOOH without test compound). 

#### 3.4.2. Cellular Antioxidant Activity (CAA) Assay

The CAA assay was modified from Wolfe and Liu [20]. HepG2 cells were seeded and grown as described for the CLPAA assay. Cells were washed with PBS between additions of new reagents. Total reaction volume was 100 μL. The incubations were at 37 °C, 5% CO_2_. Cells were then incubated with 25 μM DCFH-DA (Sigma-Aldrich) and 20 μL test compound for 1 h. After incubation, 600 μM AAPH was added and the plate immediately placed in in a Victor3 Plate Reader (excitation/emission; 485/520 nm). The plate was incubated for 1 h before the second reading. Results are presented as relative values compared to the AAPH control. 

#### 3.4.3. Cytotoxicity

Cytotoxicity was studied in HepG2, MRC-5 cells (normal human lung fibroblasts) and THP-1 cells for 2 h (HepG2), 6 h (THP-1) and 24 h (HepG2, MRC-5 and THP-1). For the 2 h study, 80,000 HepG2 cells were seeded per well. For the 24 h study, 50,000 HepG2 cells, 7500 MRC-5 cells and 10,000 THP-1 cells were used. HepG2 and MRC5 were grown over night, then washed with PBS and added 50 μL test compound at various concentrations diluted in MEM Earle’s supplemented as above but without FBS. THP-1 cells were grown as described in the immune assay. After incubation, 10 μL of CellTiter 96^®^ AQ_ueous_ One Solution Reagent (Promega, Madison, WI, USA) was added and plates were then incubated further for 1 h. Absorbance was measured at 485 nm in a DTX 880 Multimode Detector. Results were calculated as % survival compared to negative (assay media) and positive (Triton X-100; Sigma-Aldrich) control.

#### 3.4.4. Anti-Inflammatory

Approximately 10^5^ THP-1 cells supplemented with 50 ng/mL PMA were seeded in 96 well plates and incubated at 37 °C, 5% CO_2_ for 48 h. The cells were controlled after 24 h by microscopy to make sure they had started to differentiate. After 48 h, the cells were washed and new RPMI (w/o PMA) added before 24 h incubation.

The cells were then added 90 µL fresh medium and 10 µL test compound at various concentrations in duplex. Controls were included in every test run. After incubation for 1 h, all samples except negative cell controls were incubated with 1 ng/mL lipopolysaccharide (LPS; end concentration) for another 6 h at 37 °C. The reactions were stopped by freezing the plates at −80 °C immediately after incubation. 

One day prior to the ELISA testing of IL-1β and TNFα secretion, MaxiSorp 96F-well plates (Nunc) were coated with 2 µg/mL capture antibody (eBioscience, San Diego, CA, USA) and placed in the refrigerator overnight. 

Between every step, plates were washed with TBS (pH 7.4, with 0.05% Tween-20). All incubations were at room temperature with shaking. A volume of 200 µL blocking buffer (TBS w/2% BSA) was added to the plates and incubated for 1 h. TNFα samples were diluted 1:4 or 1:10 and IL-1β samples diluted 1:2. Standard concentrations of IL-1β and TNFα were added to each plate before incubation for 2 h. Biotin coupled anti-human antibody (eBioscience) was diluted in assay diluent (TBS with 1% BSA) to 3 µg/mL and added to each well and incubated for 1 h. Diluted ExtrAvidin^®^-Alkaline Phosphatase (Sigma-Aldrich) was added and plates incubated for 30 min. 100 μL pNPP substrate (Sigma-Aldrich, 1 mg/mL in 1 M diethanolamin buffer pH 9.8) was added to each well, incubated for 45 min and results read at 405 nm.

#### 3.4.5. Endotoxin Removal

We used Detoxi-Gel Endotoxin Removing Columns from Thermo Scientific (Waltham, MA, USA) according to the manufacturers’ description. Synthesized barettin was dissolved in DMSO to 100 mg/mL. The sample was further diluted in pyrogen-free water to 5 mL. After regeneration and equilibration of the column the sample was applied and the flow-through collected. The sample was then freeze-dried overnight. The test tube with barettin was weighed before and after elution to estimate the sample weight. 

## 4. Conclusions

Compounds with combined antioxidant and anti-inflammatory properties are of interest for treatment of for instance cardiovascular diseases such as atherosclerosis. Atherosclerosis is a widespread disease, especially in the western world and today there are no therapeutic treatment directly targeting this disease except cholesterol lowering drugs (statins). We found that barettin possess both anti-inflammatory and antioxidant properties making it a candidate for further studies as an atheroprotective compound. We also found interesting differences between barettin and debromobarettin, indicating that bromine could be important for *in vivo* activity of the compounds. Both compounds are active in the biochemical antioxidant assays but only barettin is active in the cellular CLPAA assay where it reduced lipid peroxidation in HepG2 cells. Barettin also inhibited IL-1β and TNFα reduction in THP-1 immune cells. Based on these results barettin is interesting as a lead compound for further structure-activity studies to elucidate the modes of action and any clinical potential.

## References

[1] Lidgren G., Bohlin L., Bergman J. (1986). Studies of swedish marine organisms VII. A novel biologically active indole alkaloid from the sponge *Geodia barretti*. Tetrahedon Lett..

[2] Sölter S., Dieckmann R., Blumenberg M., Francke W. (2002). Barettin, revisited?. Tetrahedon Lett..

[3] Johnson A.-L., Bergman J., Sjögren M., Bohlin L. (2004). Synthesis of barettin. Tetrahedon.

[4] Sjögren M., Göransson U., Johnson A.-L., Dahlström M., Andersson R., Bergman J., Jonsson P.R., Bohlin L. (2004). Antifouling activity of brominated cyclopeptides from the marine sponge *Geodia barretti*. J. Nat. Prod..

[5] Sjögren M., Johnson A.L., Hedner E., Dahlström M., Göransson U., Shirani H., Bergman J., Jonsson P.R., Bohlin L. (2006). Antifouling activity of synthesized peptide analogs of the sponge metabolite barettin. Peptides.

[6] Hedner E., Sjogren M., Hodzic S., Andersson R., Goransson U., Jonsson P.R., Bohlin L. (2008). Antifouling activity of a dibrominated cyclopeptide from the marine sponge *Geodia barretti*. J. Nat. Prod..

[7] Sjögren M., Dahlström M., Göransson U., Jonsson P.R., Bohlin L. (2004). Recruitment in the field of *Balanus improvisus* and *Mytilus edulis* in response to the antifouling cyclopeptides barettin and 8,9-dihydrobarettin from the marine sponge *Geodia barretti*. Biofouling.

[8] Hedner E., Sjögren M., Frändberg P.-A., Johansson T., Göransson U., Dahlström M., Jonsson P., Nyberg F., Bohlin L. (2006). Brominated cyclodipeptides from the marine sponge *Geodia barretti* as selective 5-HT ligands. J. Nat. Prod..

[9] Sjögren M., Jonsson P.R., Dahlström M., Lundälv T., Burman R., Göransson U., Bohlin L. (2011). Two brominated cyclic dipeptides released by the coldwater marine sponge *Geodia barretti* act in synergy as chemical defense. J. Nat. Prod..

[10] Svenson J. (2012). MabCent: Arctic marine bioprospecting in Norway. Phytochem. Rev..

[11] Kang J., Xie C., Li Z., Nagarajan S., Schauss A.G., Wu T., Wu X. (2011). Flavonoids from acai (*Euterpe oleracea* Mart.) pulp and their antioxidant and anti-inflammatory activities. Food Chem..

[12] Zimmer A.R., Leonardi B., Miron D., Schapoval E., Oliveira J.R., Gosmann G. (2012). Antioxidant and anti-inflammatory properties of *Capsicum baccatum*: from traditional use to scientific approach. J. Ethnopharmacol..

[13] Peng J., Yuan J.-P., Wu C.-F., Wang J.-H. (2011). Fucoxanthin, a marine carotenoid present in brown seaweeds and diatoms: Metabolism and bioactivities relevant to human health. Mar. Drugs.

[14] Li H., Xia N., Förstermann U. (2012). Cardiovascular effects and molecular targets of resveratrol. Nitric Oxide.

[15] Wolfe K.L., Liu R.H. (2008). Structure-activity relationships of flavonoids in the cellular antioxidant activity assay. J. Agric. Food Chem..

[16] Lü J.-M., Lin P.H., Yao Q., Chen C. (2010). Chemical and molecular mechanisms of antioxidants: Experimental approaches and model systems. J. Cell. Mol. Med..

[17] Goya L., Mateos R., Bravo L. (2007). Effect of the olive oil phenol hydroxytyrosol on human hepatoma HepG2 cells. Eur. J. Nutr..

[18] Murakami C., Hirakawa Y., Inui H., Nakano Y., Yoshida H. (2002). Effect of tea catechins on cellular lipid peroxidation and cytotoxicity in HepG2 cells. Biosci. Biotechnol. Biochem..

[19] Alía M., Ramos S., Mateos R., Granado-Serrano A.B., Bravo L., Goya L. (2006). Quercetin protects human hepatoma HepG2 against oxidative stress induced by *tert*-butyl hydroperoxide. Toxicol. Appl. Pharmacol..

[20] Wolfe K.L., Liu R.H. (2007). Cellular antioxidant activity (CAA) assay for assessing antioxidants, foods, and dietary supplements. J. Agric. Food Chem..

[21] Hofer T., Eriksen T.E., Hansen E., Varmedal I., Jensen I.-J., Hammer Andersen J., Olsen R.L., Basu S., Wiklund L. (2011). Cellular and chemical assays for discovery of novel antioxidants in marine organisms. Studies on Experimental Models.

[22] Takamatsu S., Hodges T.W., Rajbhandari I., Gerwick W.H., Hamann M.T., Nagle D.G. (2003). Marine natural products as novel antioxidant prototypes. J. Nat. Prod..

[23] Gentry C.L., Egleton R.D., Gillespie T., Abbruscato T.J., Bechowski H.B., Hruby V.J., Davis T.P. (1999). The effect of halogenation on blood-brain barrier permeability of a novel peptide drug. Peptides.

[24] Gerebtzoff G., Li-Blatter X., Fischer H., Frentzel A., Seelig A. (2004). Halogenation of drugs enhances membrane binding and permeation. ChemBioChem.

[25] Osterud B., Bjorklid E. (2003). Role of monocytes in atherogenesis. Physiol. Rev..

[26] Patel R.P., Moellering D., Murphy-Ullrich J., Jo H., Beckman J.S., Darley-Usmar V.M. (2000). Cell signaling by reactive nitrogen and oxygen species in atherosclerosis. Free Radic. Biol. Med..

[27] Greenberg M.E., Li X.-M., Gugiu B.G., Gu X., Qin J., Salomon R.G., Hazen S.L. (2008). The lipid whisker model of the structure of oxidized cell membranes. J. Biol. Chem..

[28] Stocker R., Keaney J.F. (2004). Role of oxidative modifications in atherosclerosis. Physiol. Rev..

[29] Xie C., Kang J., Burris R., Ferguson M.E., Schauss A.G., Nagarajan S., Wu X. (2011). Açaí juice attenuates atherosclerosis in ApoE deficient mice through antioxidant and anti-inflammatory activities. Atherosclerosis.

[30] Gorbet M.B., Sefton M.V. (2005). Endotoxin: The uninvited guest. Biomaterials.

[31] Lieder R., Gaware V.S., Thormodsson F., Einarsson J.M., Ng C.-H., Gislason J., Masson M., Petersen P.H., Sigurjonsson O.E. (2013). Endotoxins affect bioactivity of chitosan derivatives in cultures of bone marrow-derived human mesenchymal stem cells. Acta Biomater..

[32] Gómez-Lechón M.J., Castell J.V., Donato M.T. (2007). Hepatocytes-the choice to investigate drug metabolism and toxicity in man: *In vitro* variability as a reflection of *in vivo*. Chem. Biol. Interact..

[33] Nakamura K., Mizutani R., Sanbe A., Enosawa S., Kasahara M., Nakagawa A., Ejiri Y., Murayama N., Miyamoto Y., Torii T. (2011). Evaluation of drug toxicity with hepatocytes cultured in a micro-space cell culture system. J. Biosci. Bioeng..

[34] Benzie I.F.F., Strain J.J. (1996). The ferric reducing ability of plasma (FRAP) as a measure of “antioxidant power”: The FRAP assay. Anal. Biochem..

[35] Huang D., Ou B., Hampsch-Woodill M., Flanagan J.A., Prior R.L. (2002). High-throughput assay of oxygen radical absorbance capacity (ORAC) using a multichannel liquid handling system coupled with a microplate fluorescence reader in 96-well format. J. Agric. Food Chem..

